# Atmospheric-Pressure Conversion of CO_2_ to Cyclic Carbonates over Constrained Dinuclear Iron Catalysts

**DOI:** 10.1021/acsomega.2c02488

**Published:** 2022-07-05

**Authors:** Sreenath Pappuru, Dina Shpasser, Raanan Carmieli, Pini Shekhter, Friederike C. Jentoft, Oz M. Gazit

**Affiliations:** † Faculty of Chemical Engineering and the Grand Technion Energy Program, Technion−Israel Institute of Technology, Haifa 320003, Israel; ‡ Department of Chemical Research Support, Weizmann Institute of Science, Rehovot 76100, Israel; § Wolfson Applied Materials Research Centre, Tel Aviv University, Ramat Aviv, Tel Aviv 6997801, Israel; ∥ Department of Chemical Engineering, University of Massachusetts Amherst, Amherst, Massachusetts 01003, United States

## Abstract

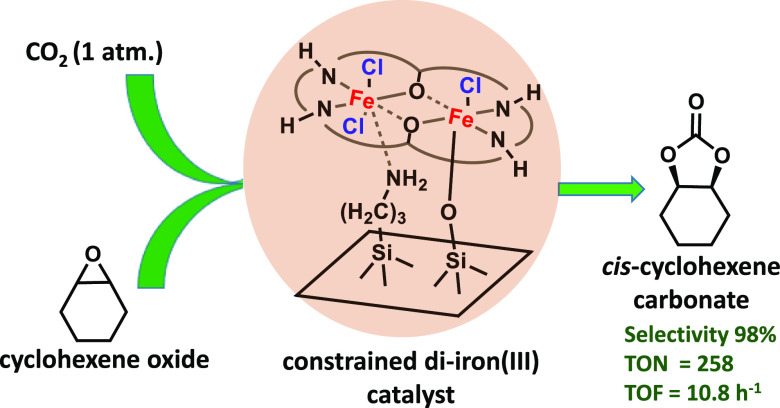

The conversion of CO_2_ and epoxides to cyclic carbonates over a silica-supported di-iron(III) complex having a reduced Robson macrocycle ligand system is shown to proceed at 1 atm and 80 °C, exclusively producing the *cis*-cyclohexene carbonate from cyclohexene oxide. We examine the effect of immobilization configuration to show that the complex grafted in a semirigid configuration catalytically outperforms the rigid, flexible configurations and even the homogeneous counterparts. Using the semirigid catalyst, we are able to obtain a TON of up to 800 and a TOF of up to 37 h^–1^ under 1 atm CO_2_. The catalyst is shown to be recyclable with only minor leaching and no change to product selectivity. We further examine a range of epoxides with varying electron-withdrawing/donating properties. This work highlights the benefit arising from the constraining effect of a solid surface, akin to the role of hydrogen bonds in enzyme catalysts, and the importance of correctly balancing it.

## Introduction

One of the greatest environmental challenges of today’s society and in the coming decades is the mitigation and utilization of anthropogenic carbon dioxide (CO_2_). If anthropogenic CO_2_ can be efficiently and sustainably converted to starting chemicals and materials, it will serve as a pivotal point for the dramatic global climate changes the world is experiencing.^1^ The coupling of CO_2_ with epoxides to cyclic carbonates is one of the promising routes for CO_2_ utilization with a 100% atom-economy.^2^ Cyclic carbonates are an important class of chemicals which have many applications such as green solvents, precursors for pharmaceuticals, fine chemicals, electrolytes in lithium-ion batteries, and precursors for biodegradable polymer synthesis.^3,4^ Industrially, the main cyclic carbonate products are propylene carbonate and ethylene carbonate.^5^ As far as we know, the most active reported catalysts for the CO_2_—epoxide coupling reactions to cyclic carbonates are homogeneous metal(salen) complexes based on Al(III), Co(II/III), or Cr(III) sites at temperatures ranging from 100 to 150 °C and CO_2_ pressures greater than 10 atm.^6−9^ Interestingly, bimetallic aluminium(salen) complexes^10,11^ and dinuclear iron(III) complexes having a reduced Robson macrocycle ligand system,^12^ in the presence of an ionic cocatalyst, were reported to be active for the synthesis of cyclic carbonates at atmospheric CO_2_ pressure. For example, the work of North et al. nicely demonstrates the conversion of various terminal epoxides into cyclic carbonates at 1 atm CO_2_ pressure with a reasonable turnover number (TON) up to 320 and a turnover frequency (TOF) value of nearly 13 h^–1^ using bimetallic aluminium(salen) complexes grafted on silica.^13−17^ With these dinuclear catalysts, the cycloaddition reaction was shown to be promoted through a dual activation mechanism via cooperative interactions of the two metal centers.^18−20^

**Figure 1:**
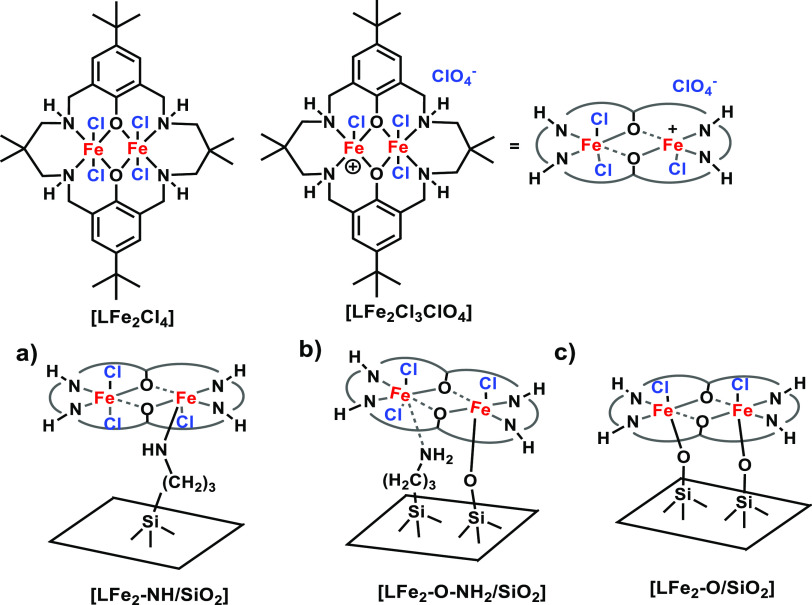
Suggested structures for silica-supported di-iron(III) species. (a) Flexible, (b) semirigid, and (c) rigid and their homogeneous analogue species (**LFe**_**2**_**Cl**_**4**_ and **LFe**_**2**_**Cl**_**3**_**ClO**_**4**_).

The heterogenization of such molecular catalysts can endow the homogeneous catalysts with attractive features such as higher stability, easy handling, easy product separation, and catalyst recovery.^21−24^ However, the presence of the solid surface, in many cases, can have configurationally constraining effects on the grafted molecular catalyst, rendering the catalytic performance less effective as compared to its homogeneous analogue.^21,22^ In the current work we demonstrate that the mode of surface grafting is key to preserve catalyst performance, and the resulting catalyst can even surpass the homogenous analogue. We do so by grafting a modified reduced Robson-type macrocycle coordinated di-iron(III) catalyst (**LFe**_**2**_**Cl**_**3**_**ClO**_**4**_) (Figure 1) using three grafting modes and evaluated their catalytic performance in the cycloaddition reaction of various epoxides with CO_2_ at a pressure of 1 atm and 80 °C.

## Results and Discussion

The full synthesis details for the modified reduced Robson-type macrocycle coordinated di-iron(III) catalyst shown in Figure 1 are provided in the Supporting Information. The molecular structure of the di-iron(III) complex (**LFe**_**2**_**Cl**_**4**_) is as reported by Williams and co-workers and is shown in Figure 1 (top left).^12^ The modified di-iron(III) complex (**LFe**_**2**_**Cl**_**3**_**ClO**_**4**_) that was used as a precursor for grafting is shown in Figure 1 (top center); its structure was validated using matrix-assisted laser desorption ionization-time of flight spectrometry (Figure S1). Using several reported grafting protocols,^25−29^ the modified complex was immobilized through interaction with either surface hydroxy or amino groups on nonporous high-surface-area silica to obtain the active solid catalysts. The di–iron complex grafting modes are shown in Figure 1: (a) tethered via a grafted propyl amine, which keeps it away from the SiO_2_ surface and hence relatively flexible (**LFe**_**2**_**-NH/SiO**_**2**_). This provides the complex with a higher configurational mobility potentially reducing steric effects for catalysis and substrate access; (b) immobilized directly through Si–OH groups to establish tight binding to the SiO_2_ surface. Given the short length of the two Si–O–Fe bonds that hold the complex close to the surface, the configurational freedom of the complex is significantly hindered and is therefore considered to be rigid (**LFe**_**2**_**-O/SiO**_**2**_); (c) immobilized through an amine and a surface silanol giving the complex a semirigid mode (**LFe**_**2**_**-O-NH**_**2**_**/SiO**_**2**_). This essentially is an intermediate state between flexible and rigid forms. Analysis of the materials using thermogravimetric analysis–mass spectrometry (TGA–MS) provided information as to the grafting loading and thermal stability (Figures S4–S6). The MS data indicate that mass loss in the range of 200–600 °C is due to the decomposition and combustion of the di–iron complex.^30^ Cross-referencing this analysis with the Fe content by inductively coupled plasma-optical emission spectrometry (ICP–OES) (Table S2), the loading of the complex in all of the catalysts was found to be similar to ∼13% wt for **LFe**_**2**_**-NH/SiO**_**2**_, ∼18% wt for **LFe**_**2**_**-O/SiO**_**2**_, and ∼15% wt for **LFe**_**2**_**-O-NH**_**2**_**/SiO**_**2**_. Interestingly, the TGA data also show that the onset temperature for decomposition increased in the following order: **LFe**_**2**_**-O/SiO**_**2**_ (224 °C), **LFe**_**2**_**-O-NH**_**2**_**/SiO**_**2**_ (243 °C), and then **LFe**_**2**_**-NH**_**2**_**/SiO**_**2**_ (252 °C). These results are consistent with a decrease in the degree of interaction of the grafted molecule with the solid support, which has been shown to promote thermal decomposition.^31,32^

Comparing the characteristic N–H vibration bands of the complex in the range of 1480–1616 cm^–1^ in the Fourier transform infrared (FTIR) spectra between the free di-iron macrocycle (Figure S5a) and the grafted materials, we clearly see significant shifts in the N–H bands following grafting (Figure S5d,e).^33,34^ In addition, the intensity of the band at 955 cm^–1^,^33^ pertaining to the Si–OH bending vibration, was found to decrease following immobilization of the complex as **LFe**_**2**_**-O-NH**_**2**_**/SiO**_**2**_ (Figure S5e) or **LFe**_**2**_**-O/SiO**_**2**_ (Figure S5d). For **LFe**_**2**_**-NH/SiO**_**2**_ (Figure S5f), the Si–OH band at ∼955 cm^–1^ only partly diminished compared to the primary amine bands at 3370 and 3305 cm^–1^ in the APS (Figure S5b). These results indicate successful binding of the **LFe**_**2**_**Cl**_**3**_**ClO**_**4**_ complex to the silica support. Analysis of the immobilized and free catalysts using diffuse reflectance (DR)-UV-vis spectroscopy showed bands at ∼200, 235, and ∼280 nm, which are related to π–π* excitation (Figures S6 and S7).^35^ An additional band, related to n−π* excitation of the macrocycle ligand, was found at ∼320 nm.^37^ Notably, in the case of APS-supported catalysts (**LFe**_**2**_**-NH/SiO**_**2**_ and **LFe**_**2**_**-O-NH**_**2**_**/SiO**_**2**_), the spectra exhibited broad absorption bands typical of grafted chiral Fe(III)-salen complexes on amine-functionalized surfaces.^36^ Consistent with the immobilization of the di–iron complex to the SiO_2_ support, we found that the UV–vis bands were slightly shifted compared to the bands of the free di–iron complex. In addition, the ligand-to-metal charge transfer transitions in all solid catalysts were centered at ∼440 nm, whereas the band for the free complex is significantly red-shifted to ∼580 nm. This serves as a strong indication that the di-iron metal centers are strongly affected by the interaction with the SiO_2_ support.^37^

**Figure 2:**
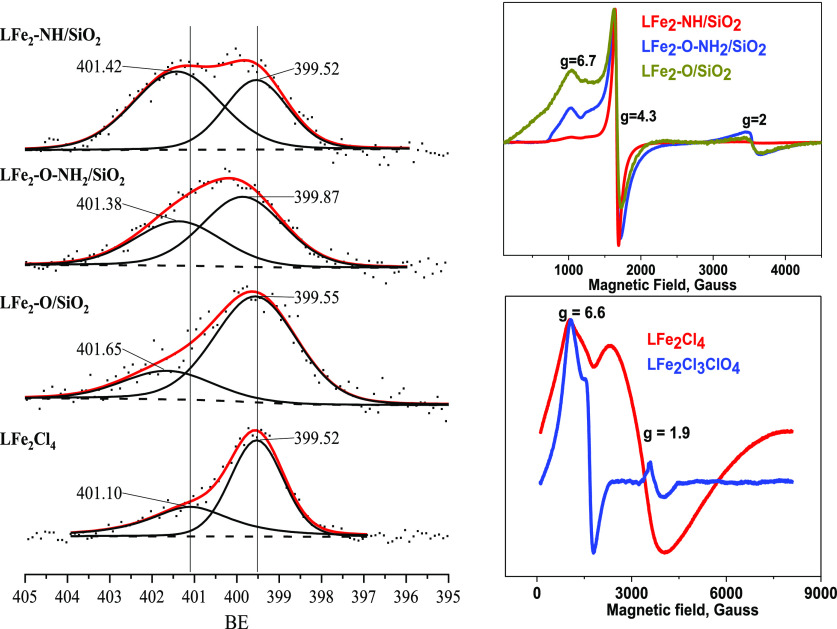
Left: XPS and right X-band EPR spectra of (a) Lfe_2_–O/SiO_2_, (b) Lfe_2_–O–NH_2_/SiO_2_, (c) Lfe_2_–NH/SiO_2_, and (d) Lfe_2_ + SiO_2_. Right: EPR spectra of di-iron(III) solid catalysts **LFe**_**2**_**-NH/SiO**_**2**_, **LFe**_**2**_**-O/SiO**_**2**_, and **LFe**_**2**_**-O-NH**_**2**_**/SiO**_**2**_ at 15 K (ν = ∼9.8 GHz). Modulation amplitude 1 G, modulation frequency 100 kHz, and MW power 2 mW.

To further probe the properties of the di-iron complex and the immobilization mode, we performed X-ray photoelectron spectroscopy (XPS). The binding energies (BEs) of Fe 2p_3/2_ and Fe 2p_1/2_ are obtained in the regions of 710 and 723 eV along with a weak satellite peak at 718.9 eV, confirming the existence of the Fe^3+^ oxidation state in all immobilized catalysts (Figure S8). Interpretation of the intensity of the O 1s signal was only possible for the free complex where the signal from the SiO_2_ support was not present (Figure S9). What was noticeable is that all immobilized catalysts showed a similar main BE at ∼532.6 shifted by −0.4 eV from the 533.0 eV O 1s BE measured for the physical mixture of the complex with bare SiO_2_ (Figure S9). This seeming shift may result from the consumption of a high binding energy component, namely SiOH, during the grafting process. Consistent with the IR data, the diminished SiOH component is the most prominent for the tight binding complex. The most insightful information was gained from the N 1s XPS spectrum (Figure 2). Fitting of the spectra reveals two distinct peaks in all samples consistent with an electron-rich and an electron-deficient N species, as detailed in Table S1. It was observed that the **LFe**_**2**_**Cl**_**4**_ reference sample showed peaks at 399.5 and 401.1 eV with a ∼2:1 area ratio. The presence of two types of N species in a symmetrical macrocycle is attributed to the coexistence of three different forms of the complex, where each had different portions of residual tetrahydrofuran (THF) (synthesis solvent) coordinated at the di-iron centers. The presence of coordinated THF species was confirmed by the analysis of the Fe, N, Cl, and O atomic composition from XPS; see schematic and detailed explanations in Tables S1–S3. Fitting of the **LFe**_**2**_**-O/SiO**_**2**_ N 1s signal showed that only the second peak upshifted by ∼0.5 eV as compared to the **LFe**_**2**_**Cl**_**4**_ sample, indicating the coordination of the di–iron complex to an electron-donating surface, that is, to Si–OH groups (Figure 2 and Table S1). Based on the atomic concentration of Fe and Cl and N as well as the N 1s peak area ratio (∼3:1), we concluded that the complex was grafted either via two Si–OH or via one Si–OH and that the two forms occurred with a 3:1 abundance (Tables S1 and S2). For the **LFe**_**2**_**-O-NH**_**2**_**/SiO**_**2**_ catalyst, the measured N 1s peak area ratio was found to be 3:2, which closely matches with the binding through both Si–OH and grafted NH_2_. The atomic concentration of Fe indicates that about 3/4 (0.73 mmol) of the NH_2_ remained unbound (Tables S1 and S2). For the **LFe**_**2**_**-NH/SiO**_**2**_ catalyst, the N 1s first peak appears at a low binding energy of 399.5 eV, which corresponds to the value seen for the **LFe**_**2**_**Cl**_**4**_ free complex (Figure 2). However, the second peak is upshifted by 0.32 eV, which is consistent with coordination of the NH_2_ group; see Table S1. This latter conclusion is supported by the N 1s 1.54:1 peak ratio (Table S1), which confirms exclusive grafting through the NH_2_ groups in contrast to the case of **LFe**_**2**_**-O-NH**_**2**_**/SiO**_**2**_.

It can be expected that the different immobilization modes, depicted in Figure 1, will be evident in changes to the coordination environment of the dinuclear iron.^38−40^ To examine the coordination, we recorded the electron paramagnetic resonance (EPR) spectra at 15 K for all catalysts (Figure 2) (right). The CW-EPR clearly differentiates between the three grafted modes. The EPR spectrum of the flexible **LFe**_**2**_**-NH/SiO**_**2**_ catalyst exhibits a dominant signal at *g* = 4.3 and almost no signals at *g* = 6.7 and *g* = 2.0, which indicates that the two Fe^III^ in **LFe**_**2**_**-NH/SiO**_**2**_ are in the same environment and are high spin (hs) ferric ions (hs-Fe^III^). On the other hand, the rigid **LFe**_**2**_**-O/SiO**_**2**_ and semirigid **LFe**_**2**_**-O-NH**_**2**_**/SiO**_**2**_ catalysts displayed two hs Fe^III^, species, one with *g* = 4.3 and a second one with *g*_⊥_ = 6.7 and *g*_||_ = 2, indicating the presence of two different Fe III environments. However, the semirigid **LFe**_**2**_**-O-NH**_**2**_**/SiO**_**2**_ catalyst showed a strong EPR signal at *g* = 4.3 and a less dominant signal at *g*_⊥_ = 6.7 and *g*_||_ = 2, which is a superposition of the first two catalysts, indicating the presence of the two binding modes. These observations are consistent with the conclusions drawn from the XPS data. In sum, the above results clearly show that the different grafting protocols resulted in the formation of different binding modes. Presumably, the tight binding of the complex to the surface in **LFe**_**2**_**-O/SiO**_**2**_ makes for a relatively rigid structure. The grafting through the amine only in **LFe**_**2**_**-NH/SiO**_**2**_ creates loose binding rendering the complex more flexible, whereas binding through both amine and hydroxy generates a semirigid complex.

Reaction testing of these catalysts was conducted in neat epoxide at 80 °C and under 1 or 15 atm of CO_2_ with 2 equivalents of bis(triphenylphosphino)iminium chloride ([PPN]Cl). As reported and verified here, under the current reaction conditions, PPNCl was inactive on its own.^11^ In addition, all catalysts were found to be inactive without the addition of PPNCl. PPNCl is a bulky cationic initiator, which acts to labialize the metal-nucleophile bond of either the Cl or carbonate to promote the rate-limiting, epoxide ring-opening step or to facilitate the ring closure (backbiting) step.^11^ A proposed mechanism with respect to the current active catalyst is shown in Figures S10 and S11. The catalysts were initially evaluated for CO_2_ conversion using the relatively bulky cyclohexene oxide (CHO) as the coupling agent, which is less active for ring-opening as compared to other epoxides due to its bicyclic nature. The selectivity to the cyclic carbonate was generally >90%, and the isolated cyclic carbonate yield was used to determine TON and TOF. The catalysis results at 1 atm of CO_2_ show that the semirigid **LFe**_**2**_**-O-NH**_**2**_**/SiO**_**2**_ catalyst was more active (TOF of 10.8 h^–1^) as compared to the rigid **LFe**_**2**_**-O/SiO**_**2**_ (TOF = 8.3 h^–1^) and the flexible **LFe**_**2**_**-NH/SiO**_**2**_ (TOF = 5.6 h^–1^) catalysts (Tables 1 and S4). This finding is consistent with what is known for enzyme-inspired synthetic catalysts, which show enhanced performance when able to balance between the flexibility needed for correct catalyst alignment and the rigidity of the support required for stability of the active sites^34^ and accessibility of the substrate.^41,42^ Notably, the semirigid **LFe**_**2**_**-O-NH**_**2**_**/SiO**_**2**_ catalyst exhibited higher activity as compared to the homogeneous counterparts, whereas the rigid and flexible showed similar or diminished performance (Table 1 entries 1 to 5). As far as we know, this is the first example using CHO as the coupling agent, which shows this transformation to proceed with good yields using a heterogeneous metal-based catalyst under 1 atm CO_2_. In a few rare examples, non-metal-based heterogeneous catalysts have been used for CHO transformation under 1 atm CO_2_ (see Table S6).

**Table 1:** Conversion of CO_2_ to Cyclic Carbonates Using Di-Iron(III) Solid Catalysts^a^

entry	catalysts	yield (g/mg_cat_)^b^	TON^c^	TOF^d^/h^–1^	CHC^e^ %
1	LFe_2_Cl_4_ (homogenous)	0.346 ± 0.004	195	8.1	98
2	LFe_2_Cl_3_ClO_4_ (homogenous)	0.355 ± 0.003	217	9.0	98
3	LFe_2_-NH/SiO_2_ (flexible)	0.223 ± 0.007	135	5.6	93
4	LFe_2_-O/SiO_2_ (rigid)	0.327 ± 0.01	199	8.3	95
5	LFe_2_-O-NH_2_/SiO_2_ (semirigid)	0.427 ± 0.005	258	10.8	98
6	LFe_2_Cl_3_ClO_4_+APS	0.256 ± 0.007	156	6.5	91
7	LFe_2_Cl_3_ClO_4_+SiO_2_	0.301 ± 0.007	184	7.7	95

a Reactions were carried out under neat epoxide at a loading of [di-iron(III) cat.]/PPNCl/cyclohexene oxide of 1:2:1000. For full experimental details, see the Supporting Information.

b Measured weight of the isolated cyclic carbonate (yields were normalized per 10 mg of catalyst).

c TON = number of moles of cyclohexene oxide consumed/number of moles of [di-iron(III) cat].

d TOF = TON/reaction time.

e Determined by ^1^H NMR spectroscopy.

**Figure 3:**
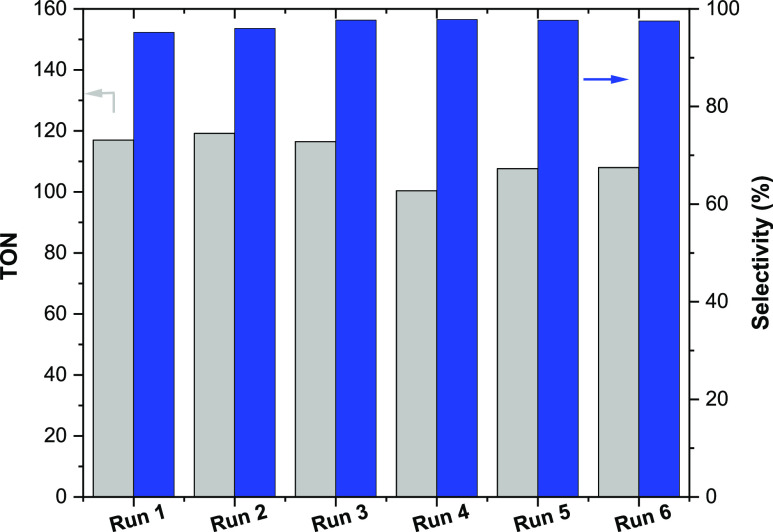
Recyclability studies of **LFe**_**2**_**-O-NH**_**2**_**/SiO**_**2**_ for the cycloaddition of CO_2_ to cyclohexene oxide under mild conditions (80° C, 1 atm, 12 h). The Fe leaching was measured by ICP–OES and found to reduce with cycles in the following order: 1st run 5.3%; 2nd run 3.5%; 3rd and 4th runs 3%; 5th and 6th runs 2%. Note: TON was calculated with respect to the amount of Fe in each cycle.

Reaction product analysis using ^1^H NMR showed that for all catalysts, the methyne protons had a chemical shift of 4.64 ppm (versus 3.90 ppm for the *trans*-CHC), which means that the *cis*-cyclohexene carbonate (*cis*-CHC) was exclusively produced (Figure S12).^11^ Only few reported homogeneous catalysts show the formation of the *cis*-CHC product by the coupling with CO_2_.^12,43,44^ Further testing was done for the semirigid **LFe**_**2**_**-O-NH**_**2**_**/SiO**_**2**_ catalyst with various epoxide substrates (Table 2 and Figures S13–S16). We further found that the epoxides having stronger electron-withdrawing groups gave higher TOF in contrast to electron-rich epoxides. This trend can be observed by the obtained yield, TON and TOF, which decreased in the following order: phenyl glycidyl ether (PGE) > styrene oxide (SO) > *tert*-butyl glycidyl ether (TBGE) > CHO. As can be expected, upon increasing the CO_2_ pressure to 15 atm at 80 °C, the yield and TOF for cyclic carbonate production significantly increased giving TOF values close to 37 (Table S4). As shown in Figure 3, the semirigid **LFe**_**2**_**-O-NH**_**2**_**/SiO**_**2**_ catalyst could be reused, following a washing step, at least 6 times without significant loss in TON or change to selectivity, Figure 3.

**Table 2:** Synthesis of Cyclic Carbonates from Various Epoxides Using LFe_2_-O-NH_2_/SiO_2_^a^

entry	epoxide	*T*/^o^C	% epoxide conversion^b^	TOF^c^/h^–1^	CHC^b^%
1	PO	30	29	18.6	99
2	CHO	80	18.2	10.8	98
3	TBGE	80	41	17.1	99
4	SO	80	65	25.5	99
5	PGE	80	89	37.0	99

a Reaction conditions: neat epoxide, 1 atm CO_2_ 24 h, at a loading of [di-iron(III) cat.]/PPNCl/epoxide of 1:2:1000.

b Determined by measuring the weight of the isolated cyclic carbonate.

c TOF = TON/h.

In conclusion, the current work describes the synthesis, characterization, and catalytic testing of a modified SiO_2_ immobilized di–iron complex. The combined results by XPS, EPR, TGA–MS, DRIFTS, and DR-UV-vis spectroscopy show three distinct immobilization modes, namely, flexible, semirigid, and rigid. Catalytic testing demonstrates that the catalyst in the semirigid mode (**LFe**_**2**_**-O-NH**_**2**_**/SiO**_**2**_) outperformed both the flexible (**LFe**_**2**_**-NH/SiO**_**2**_) and rigid (**LFe**_**2**_**-O/SiO**_**2**_) modes and the homogeneous catalyst in the conversion of CO_2_ and epoxides to cyclic carbonates. The semirigid catalyst mode was shown to have markedly high activity, exhibiting high TON values in the range of 100–800 and TOF values close to 37 h^–1^ for cyclic carbonates synthesis at 1 atm CO_2_. This is remarkable given that other examples in the literature including microporous organic network systems,^45−47^ MOFs,^48^ and SiO_2_ supported di-aluminum complexes^13−17^ have lower TON at 1 atm CO_2_ or require the presence of tetraalkylammonium salts and CO_2_ pressures greater than 10 atm to reach similar TON values (Tables S5 and S6 in Supporting Information). We further demonstrate that the semirigid catalyst was reused up to six times without significant loss in TON or change in selectivity. As far as we know, this is the only example of a recyclable heterogeneously catalyzed *cis*-cyclohexene carbonate synthesis at 1 atm CO_2_.

## Supporting Information Available

Detailed experimental section, data for MS, ^1^H-NMR, proposed reaction cycles, XPS and UV–vis, FTIR, and TGA–MS analyses (PDF)
