# Supplementary material for: Atmospheric-Pressure Conversion of CO2 to Cyclic Carbonates over Constrained Dinuclear Iron Catalysts
Source: ACS Omega. 2022 Jul 5;7(28):24656–61. doi: 10.1021/acsomega.2c02488 (PMC9301958; doi:10.1021/acsomega.2c02488)
Supplement: Supplementary file 1 — ao2c02488_si_001.pdf [file ao2c02488_si_001.pdf]

−Supporting Information for the manuscript entitled−

"Atmospheric-Pressure Conversion of CO_2 to Cyclic Carbonates over Constrained Dinuclear Iron Catalyst"

Sreenath Pappuru,[a] Dina Shpasser,[a] Raanan Carmieli,[b] Pini Shekhter,[c] Friederike C. Jentoft,[d] and Oz M. Gazit*[a]

[a] Faculty of Chemical Engineering, Technion−Israel Institute of Technology, Haifa 320003, Israel.

[b] Department of Chemical Research Support, Weizmann Institute of Science, Rehovot 76100, Israel.

[c] Wolfson Applied Materials Research Centre, Tel Aviv University, Ramat Aviv, Tel Aviv 6997801, Israel.

[d] Department of Chemical Engineering, University of Massachusetts Amherst, Amherst, MA 01003, USA.

Corresponding author: *ozg@technion.ac.il*

Table of Contents

1. Experimental 2

1.2 Synthesis Procedures: 3

1.3 Typical procedure for the synthesis of cyclic carbonate from an epoxide and 1 atm CO_2 4

1.4 Characterization of cis-cyclic carbonate products: 5

2. Supplementary Figures and Tables 6

Figure S1. Mass spectrum (MALDI-TOF) of $LFe_2Cl_3ClO_4$. 6

Figure S2. TGA-MS analysis of flexible di-nuclear solid catalyst **LFe_2-NH/SiO_2**. Dashed line marks the 252 °C onset for complex decomposition. 7

Figure S3. TGA-MS analysis of semi-rigid di-nuclear solid catalyst **LFe_2-O-NH_2/SiO_2**. Dashed line marks the 243 °C onset for complex decomposition. 7

Figure S4. TGA-MS analysis of rigid di-nuclear solid catalyst **LFe_2-O/SiO_2**. Dashed line marks the 224 °C onset for complex decomposition. 8

Figure S5. FT-IR spectra of (a) $LFe_2Cl_3ClO_4$ (b) APS (c) Aerosil-200 (d) LFe_2-O/SiO_2 (e) LFe_2-O-NH_2/SiO_2 (f) LFe_2-NH/SiO_2. 9

Figure S6. UV-vis diffuse reflectance spectra of: (a) **LFe_2Cl_4** (black line), **(b) $Lfe_2Cl_3ClO_4$** (blue line) and (c) **Lfe_2-O/SiO_2** (red line). 10

Figure S7. UV-vis spectra of: (a) **$LFe_2Cl_3ClO_4$** (blue line), **(b) LFe_2-O-NH_2/SiO_2** (purple line) and (c) **LFe_2-NH/SiO_2** (green line). 10

Table S1. Interpretation of proposed models by comparing the experimental and theoretical peak areas from XPS. 11

Table S2. Atomic composition for solid catalysts based on HR-XPS analysis 11

Table S3. LFe_2Cl_4 homogeneous complex molecular determination based on Fe, N, Cl and O atomic composition from HR-XPS analysis[a] 12

Figure S8. XPS analysis of Fe2p binding energy regions in di-iron (III) solid catalysts. Spectra are offset for clarity. 12

Figure S9. XPS analysis of O1s binding energy regions in di-iron (III) solid catalysts. 13

Table S4. Conversion of CO_2 to cyclic carbonates using di-iron(III) catalysts at a CO_2 pressure of 15 atm and a temperature of 80 ^oC.[a] 13

Figure S10. Illustrates the proposed pathways for formation of active anionic species, iron-alkoxide species and iron-carbonate species *via* dual-activation mechanism by two iron metal centers. 14

Figure S11. Backbiting reactions from carbonate and alkoxide to form *cis*-cyclohexene carbonate and *trans*-cyclohexene carbonate respectively.[3] 14

Table S5: Catalytic activity of different heterogeneous catalysts for cycloaddition of CO2 with terminal epoxides 15

Table S6: Catalytic activity of different heterogeneous catalysts for cycloaddition of CO2 with cyclohexene oxide 16

Figure S12. ^1H NMR spectrum ($CDCl_3$, 400 MHz, 298 K) of *cis*-cyclic cyclohexene carbonate (CHC) derived by using LFe_2-O-NH_2/SiO_2 and [PPN]Cl at 1atm CO_2. LFe_2-O-NH_2/SiO_2 /PPNCl/epoxide = 1:2:1000, Table 1 entry 5. 17

Figure S13. ^1H NMR spectrum ($CDCl_3$, 400 MHz, 298 K) of propylene carbonate (PC) derived by using LFe_2-O-NH_2/SiO_2 and [PPN]Cl at 1atm CO_2. LFe_2-O-NH_2/SiO_2/PPNCl/epoxide = 1:2:1000, Table 2 entry 1). 18

Figure S14. ^1H NMR spectrum ($CDCl_3$, 400 MHz, 298 K) of phenyl glycidyl cyclic carbonate (PGCC) derived by using LFe_2-O-NH_2/SiO_2 and [PPN]Cl at 1atm CO_2. LFe_2-O-NH_2/SiO_2 /PPNCl/epoxide = 1:2:1000, Table 2 entry 4. 18

Figure S15. ^1H NMR spectrum ($CDCl_3$, 400 MHz, 298 K) of *tert*-butyl glycidyl cyclic carbonate (TGCC) derived by using LFe_2-O-NH_2/SiO_2 and [PPN]Cl at 1atm CO_2. LFe_2-O-NH_2/SiO_2 /PPNCl/epoxide = 1:2:1000, Table 2 entry 3. 19

Figure S16. ^1H NMR spectrum ($CDCl_3$, 400 MHz, 298 K) of styrene carbonate (SC) derived by using LFe_2-O-NH_2/SiO_2 and [PPN]Cl at 1atm. CO_2. LFe_2-O-NH_2/SiO_2/PPNCl/epoxide = 1:2:1000, Table 2 entry 2 19

Figure S17. The reaction setup used for the conversion of CO_2 to cyclic carbonate under 1 atm CO_2 pressure. 20

3. **References:** 21

1. Experimental

1.1 Materials and Methods

The pro-ligand (H_2L) and di-iron complex were prepared according to literature procedures.[1,2] The chemicals required for all these syntheses were purchased from Aldrich and used as received unless

otherwise noted. The deuterated solvent for NMR studies, *i.e.* $CDCl_3$ was acquired from Aldrich and used as received. The common reagents and common solvents were acquired locally and used as received. All ^{1}H and ^{13}C NMR spectra were recorded on a Bruker Avance 400 MHz spectrometer at 25 ^{o}C with chemical shifts given in parts per million (ppm) using the residual solvent peak as reference, located at 7.26 ppm in the case of $CDCl_3$ and at 2.5 ppm in the case of d_6-DMSO. Fourier transform infrared spectra (FTIR) were recorded on a Nicolet 8700 FTIR spectrometer equipped with an attenuated total reflection (ATR) stage. ICP-OEM analyses were done on a Thermo Scientific ICP Spectrometer ICAP 6300 DUO. Samples were digested in 1 ml conc. HCl by stirring at 65 °C for 12 h, and the resulting solution was diluted with deionized water prior to ICP-OES analysis. TGA-MS measurements were performed on a SETARAM Labsys-Evo coupled to a Hiden QGA-pro using synthetic air as the carrier gas (20% O_2 in Ar, 30 ml/min, 5 °C/min). High resolution X-ray photoelectron spectroscopy (HR-XPS) measurements were performed in an analysis chamber (UHV – $2*10^{-10}$ Torr during analysis) using a Versaprobe III – PHI Instrument (PHI, USA). The sample was irradiated with a Focused X-Ray AlKα monochromated X-ray source (1486.6eV) using an X-ray beam (size 200 micron, 50W, 15 kV). The outcoming photoelectrons were directed to a Spherical Capacitor Analyzer (SCA). The sample charging was compensated by a Dual Beam charge neutralization based on a combination of a traditional electron flood gun and a low energy argon ion beam. Diffuse reflectance UV-vis spectra were recorded on a Agilent Cary 5000 UV-vis spectrophotometer with a DRA-2500 integrating sphere. Electron paramagnetic resonance (EPR) spectra were obtained on a Bruker EMX EPR spectrometer controlled with a Bruker ER 041 XG microwave bridge at 15 or 77 K. The EPR spectrometer was equipped with a continuous-flow liquid He cryostat and an ITC503 temperature controller made by Oxford Instruments, Inc.

1.2 Synthesis Procedures:

Synthesis of LFe_2Cl_4:

Reduced Robson macrocycle ligand (**LH_2**)[1] and the corresponding di iron (III) complex (**LFe_2Cl_4**)[2] were synthesized by slight modification of reported procedure. The clear dry THF solution of ligand **LH_2** (933 mg, 1.69 mmol) in dry flask was kept in a glove box freezer for 0.5 h. KH (147 mg, 3.4 mmol) was slowly added by small portions to this cold THF solution. After 12 h of stirring, the reaction mixture was centrifuged, then [$FeCl_3$(DME)] (860 mg, 3.4 mmol) was added to this colorless solution, and immediately the reaction mixture changed to dark blue. The resulting mixture was left to stir at RT for 24 h. The white KCl precipitates were eliminated by centrifugation, and the dark blue THF supernatant was removed in vacuum. The dark blue solid residue was finally washed with hexane (3 × 15 mL), then dried under vacuum for 24 h. (yield: 1.3 g, 96 %). Elemental Anal. for $C_{42}H_{70}Cl_4Fe_2N_4O_4$: Calculated; C, 53.18; H, 7.44; N, 5.91; O, 6.75 Found; C, 52.55; H, 7.11; N, 6.23; O, 5.91.

Synthesis of $LFe_2Cl_3ClO_4$:

Calculated amount of **LFe_2Cl_4** (2.26 g, 28 mmol) was dissolved in dry THF (30 mL) and transferred into a Schlenk tube containing silver perchlorate (0.6 g, 28.6 mmol) in dry acetonitrile (70 mL) and stirred at 65 ^{o}C for 24 h. After the reaction, the obtained dark blue solution was filtered through Celite and washed with acetonitrile. The filtrate was concentrated and dried under vacuum (yield: 2.3 g, 94 %). Molecular cation (MALDI-TOF): ~849 gmol^{-1} [$LFe_2Cl_3ClO_4$–$1CH_3$–4H]$^+$. The sample was dissolved in TA30 (30:70 acetonitrile:TFA of 0.1% in water), drop casted on a HCCA (alfa cyano 4-hydroxy cinnamic acid) matrix and analyzed on a Bruker MALDI autoflex speed.

Elemental Anal. for $C_{34}H_{54}Cl_4Fe_2N_4O_6$: Calculated; C, 47.03; H, 6.27; N, 6.45; O, 11.06 measured: C, 47.18; H, 6.55; N, 6.27; O, 11.42.

Synthesis of LFe_2-NH/SiO_2:

A clear solution of **$LFe_2Cl_3ClO_4$** (755 mg, 0.87 mmol) in 20 mL of dry acetonitrile was transferred into a Schlenk tube and to this a suspension of 3-aminopropyl silica (0.7g) in 15mL acetonitrile was added. The reaction mixture was vigorously stirred at 85 ^oC for 48 h under argon. The resulting solid product was separated by centrifugation and washed seven times with fresh acetonitrile, three times THF, two times diethyl ether and the obtained solid product was dried under vacuum (yield: 0.589 g).

Synthesis of LFe_2-O/SiO_2:

A solution of N, N-diisopropylethylamine (112 mg, 0.87 mmol) in 5 mL of dry acetonitrile was transferred into a Schlenk tube containing a suspension of silica support ($SiO_{2\text{-}200}$, 0.7 g) in 10 mL acetonitrile, and the resulting mixture was stirred for two hours at room temperature. To this, the clear solution of **$LFe_2Cl_3ClO_4$** (755 mg, 0.87 mmol) in 20 mL of acetonitrile was added. The reaction mixture was vigorously stirred at 85 ^oC for 48 h under argon. The resulting solid product was purified in the same manner as above (yield: 0.625 g).

Synthesis of LFe_2-O-NH_2/SiO_2:

A solution of N, N-diisopropylethylamine (224 mg, 1.74 mmol) in 5 mL of dry acetonitrile was transferred into a Schlenk tube containing a suspension of 3-aminopropyl silica (0.7 g) in 10 mL acetonitrile, and the resulting mixture was stirred for two hours at room temperature. To this, the clear solution of **$LFe_2Cl_3ClO_4$** (755 mg, 0.87 mmol) in 20 mL of acetonitrile was added. The reaction mixture was vigorously stirred at 85 ^oC for 48 h under argon. The resulting solid product was purified in the same manner as above (yield: 0.611 g).

1.3 Typical procedure for the synthesis of cyclic carbonate from an epoxide and 1 atm CO_2

The catalytic conversion of CO_2 to cyclic carbonate was carried out in a 25 mL glass flask refluxing with a CO_2 balloon. In a typical catalytic cycloaddition, dry cyclohexene oxide (CHO, 9.2 mmol) and the dry di iron (III) catalyst **LFe_2-O-NH_2/SiO_2** (0.036 g, 9.2×10^{-3} mmol, according to the amount of **$LFe_2Cl_3ClO_4$**) followed by bis(triphenylphosphino)iminium chloride (PPNCl) (10.6 mg, 18.4×10^{-3} mmol) were placed in a 25 mL glass flask equipped with a magnetic stirrer. After being sealed, three times the flask was carefully evacuated and refilled with CO_2. After that, the reaction was carried out at 80 ^oC temperature by refluxing with a CO_2 balloon for the desired period of time. After the reaction, the pressure was released and the resulting mixture was diluted with CH_2Cl_2. After which, the reaction mixture was centrifuged to remove the solid catalyst and evaporated and dried in *vacuo* overnight. The product was analyzed by ^1H NMR spectroscopy without further purification as the vacuum was sufficient to remove unreacted cyclohexene oxide. Turn-over-number (TON) was calculated as [number of moles of epoxide consumed based on epoxide conversion/moles of solid catalyst]. These number were obtained by: [(isolated cyclic carbonate yield – weight of PPNCl) /142.1]/moles of solid catalyst. The calculated error is based on three separate reaction runs.

Typical procedure for the synthesis of cyclic carbonate from an epoxide and 15 atm. CO_2

For high-pressure cycloaddition reactions, the di-iron (III) catalyst **LFe_2-O-NH_2/SiO_2** (0.036 g, 9.2×10^{-3} mmol), PPNCl (10.6 mg, 18.4×10^{-3} mmol) and cyclohexene oxide (CHO, 0.092 mol) were placed in a 25 mL stainless-steel Parr reaction vessel (which was dried in an oven at 140 ºC overnight) under argon. After being sealed, the reactor was carefully evacuated and refilled with CO_2. The reaction was carried out at the specified temperature and CO_2 pressure for the desired period of time. After the reaction, the reactor was cooled in an ice-water bath and slowly depressurized. The product purification was carried out in the same manner as above.

1.4 Characterization of cis-cyclic carbonate products:

Cyclohexene carbonate (CHC)

^{1}H NMR (400 MHz, $CDCl_3$, ppm): δ = 4.68 (m, 2H, $OC\mathit{H}CH_2CH_2$), 1.89 (m, 4H, $OCHC\mathit{H}_2CH_2$), 1.61 (m, 2H, $OCHCH_2C\mathit{H}_2$), 1.42 (m, 2H, $OCHCH_2C\mathit{H}_2$).

Propylene carbonate (PC)

^{1}H NMR (400 MHz, $CDCl_3$, ppm): δ = 4.85 (m, 2H, $OCH_2C\mathit{H}CH_3$), 4.55 (t, 1H, $OCH_2C\mathit{H}CH_3$), 4.02 (m, 1H, $OC\mathit{H}_2CHCH_3$), 1.48 (m, 3H, $OCH_2CHC\mathit{H}_3$).

Styrene carbonate (SC)

^{1}H NMR (400 MHz, $CDCl_3$, ppm): δ = 5.6 (t, 1H, $OCH_2C\mathit{H}C_6H_5$), 4.72 (t, 1H, $OC\mathit{H}_2CHC_6H_5$), 4.27 (t, 1H, $OC\mathit{H}_2CHC_6H_5$), 7.21-7.36 (m, 5H, $OCH_2CHC_6\mathit{H}_5$).

Phenyl glycidyl cyclic carbonate (PGCC)

^{1}H NMR (400 MHz, $CDCl_3$, ppm): δ = 5.0 (m, 1H, $OCH_2C\mathit{H}CH_2OC_6H_5$), 4.6 (t, 1H, $OC\mathit{H}_2CHCH_2OC_6H_5$), 4.5 (m, 1H, $OC\mathit{H}_2CHCH_2OC_6H_5$), 4.2 (m, 1H, $OCH_2CHC\mathit{H}_2OC_6H_5$), 4.1 (m, 1H, $OCH_2CHC\mathit{H}_2OC_6H_5$), 7.3 (m, 2H, $OCH_2CHCH_2OC_6\mathit{H}_5$), 7.0 (t, 1H, $OCH_2CHCH_2OC_6\mathit{H}_5$), 6.9 (t, 2H, $OCH_2CHCH_2OC_6\mathit{H}_5$).

***tert*-butyl glycidyl cyclic carbonate (TGCC)**

^{1}H NMR (400 MHz, $CDCl_3$, ppm): δ = 4.7 (m, 1H, $OCH_2C\mathit{H}CH_2OC(CH_3)_3$), 4.4 (t, 1H, $OC\mathit{H}_2CHCH_2OC(CH_3)_3$), 4.3 (m, 1H, $OC\mathit{H}_2CHCH_2OC(CH_3)_3$), 3.6 (m, 1H, $OCH_2CHC\mathit{H}_2OC(CH_3)_3$), 3.5 (m, 1H, $OCH_2CHC\mathit{H}_2OC(CH_3)_3$), 1.2 (s, 9H, $OCH_2CHCH_2OC(C\mathit{H}_3)_3$).

2. Supplementary Figures and Tables

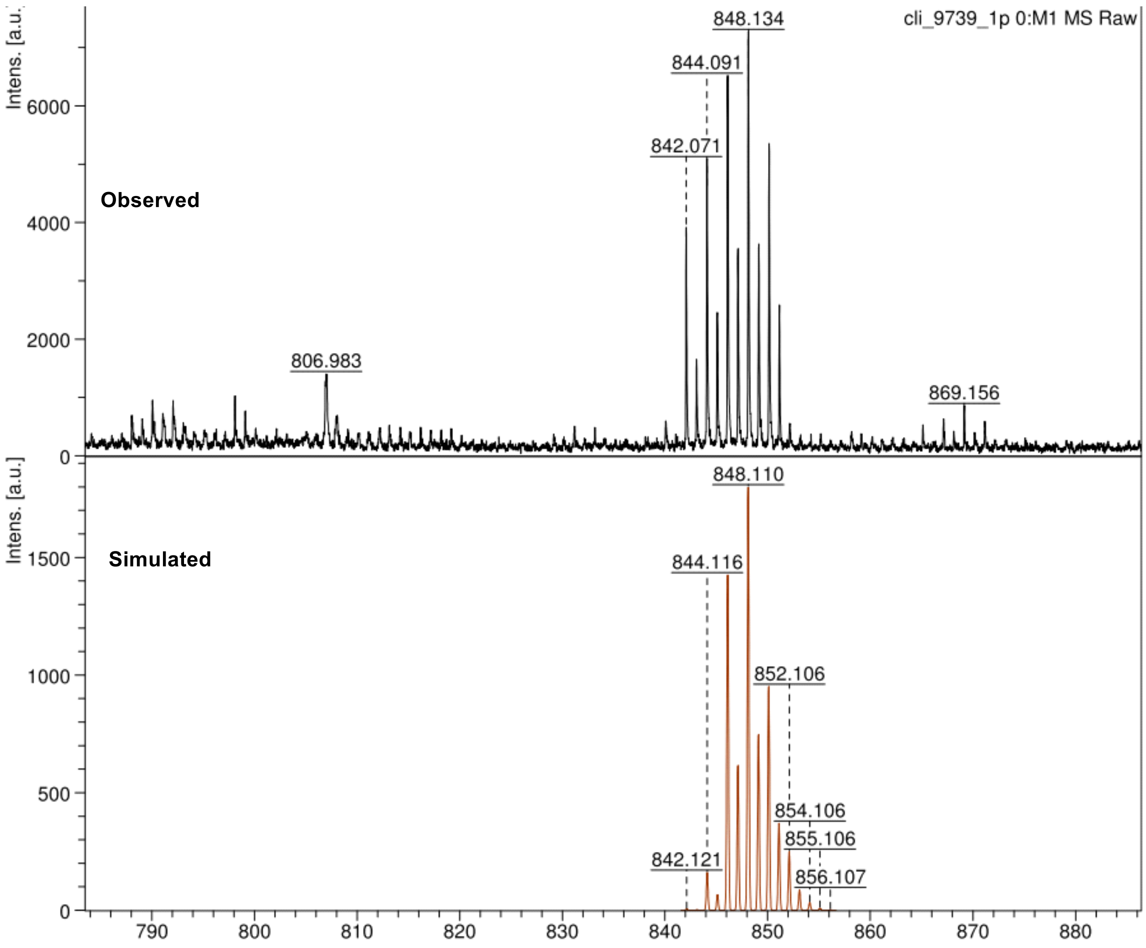

Figure S1. Mass spectrum (MALDI-TOF) of $LFe_2Cl_3ClO_4$.

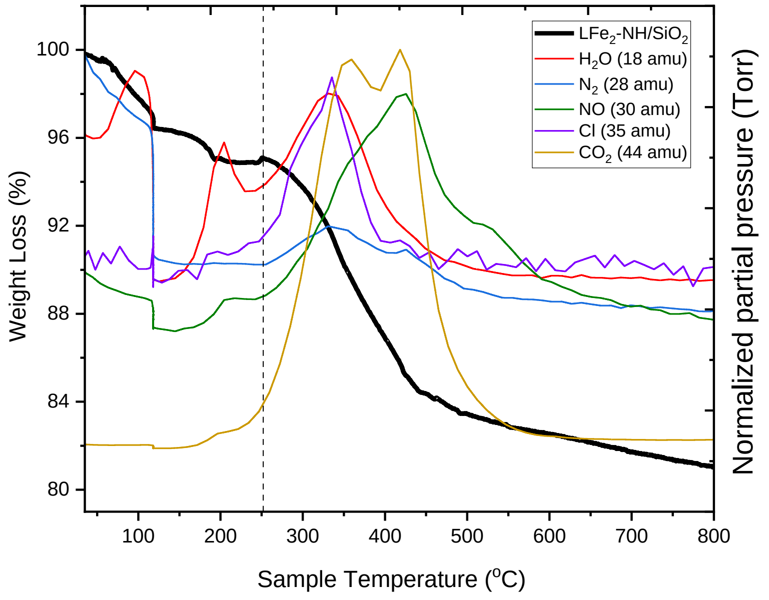

Figure S2. TGA-MS analysis of flexible di-nuclear solid catalyst **LFe_2-NH/SiO_2**. Dashed line marks the 252 °C onset for complex decomposition.

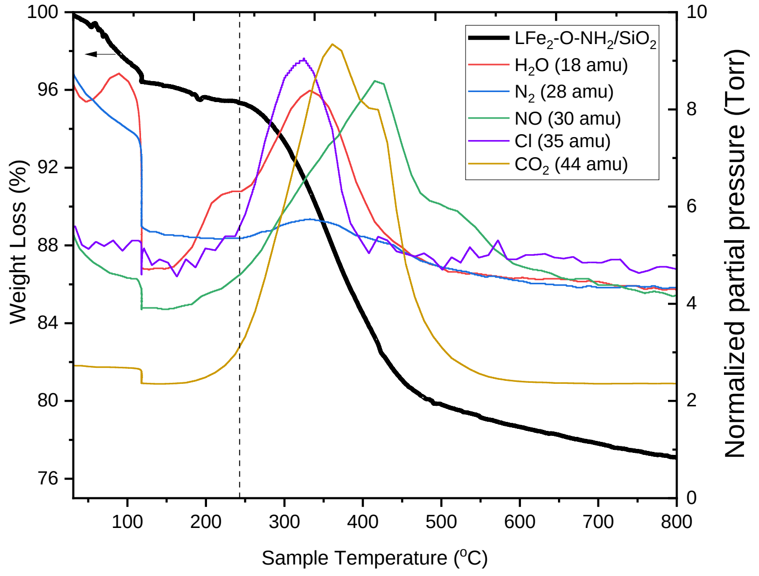

Figure S3. TGA-MS analysis of semi-rigid di-nuclear solid catalyst **LFe_2-O-NH_2/SiO_2**. Dashed line marks the 243 °C onset for complex decomposition.

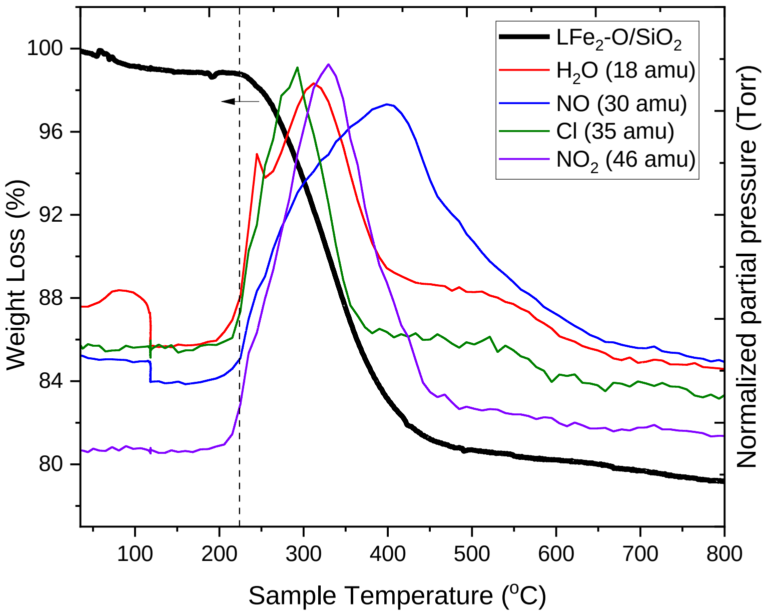

Figure S4. TGA-MS analysis of rigid di-nuclear solid catalyst **LFe_2-O/SiO_2**. Dashed line marks the 224 °C onset for complex decomposition.

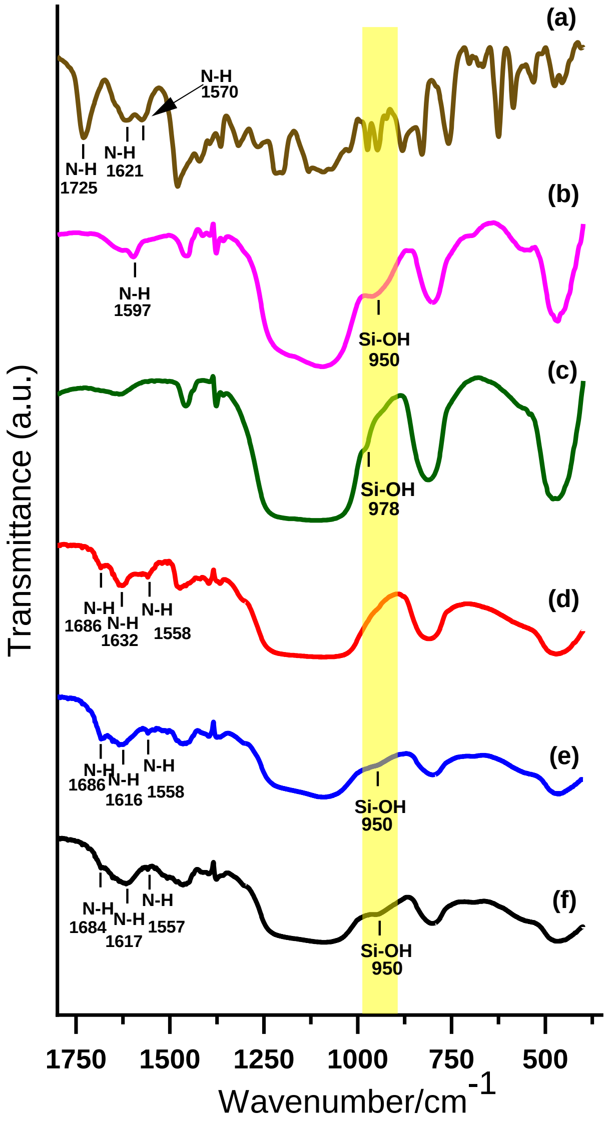

Figure S5. FT-IR spectra of (a) $LFe_2Cl_3ClO_4$ (b) APS (c) Aerosil-200 (d) LFe_2-O/SiO_2 (e) LFe_2-O-NH_2/SiO_2 (f) LFe_2-NH/SiO_2.

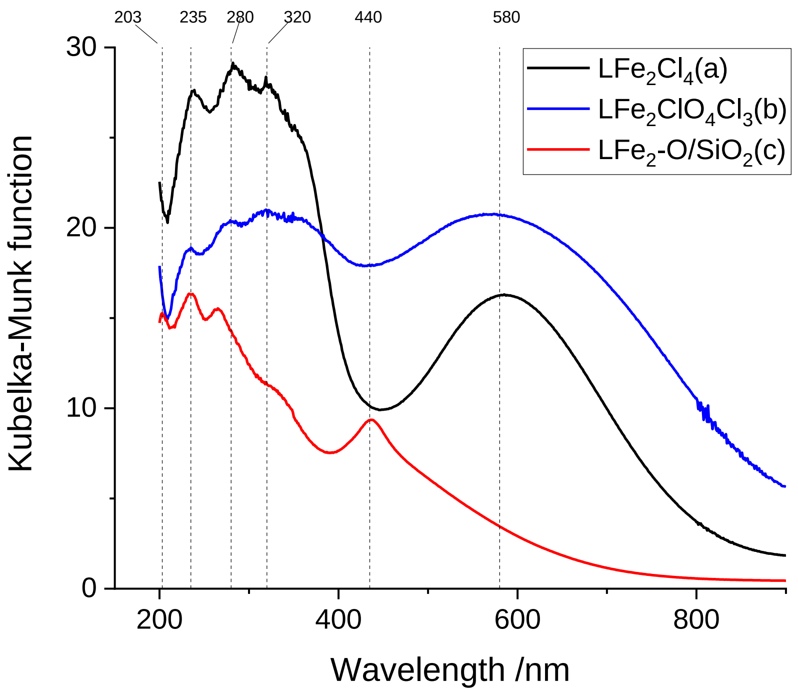

Figure S6. UV-vis diffuse reflectance spectra of: (a) **LFe_2Cl_4** (black line), **(b) $Lfe_2Cl_3ClO_4$** (blue line) and (c) **Lfe_2-O/SiO_2** (red line).

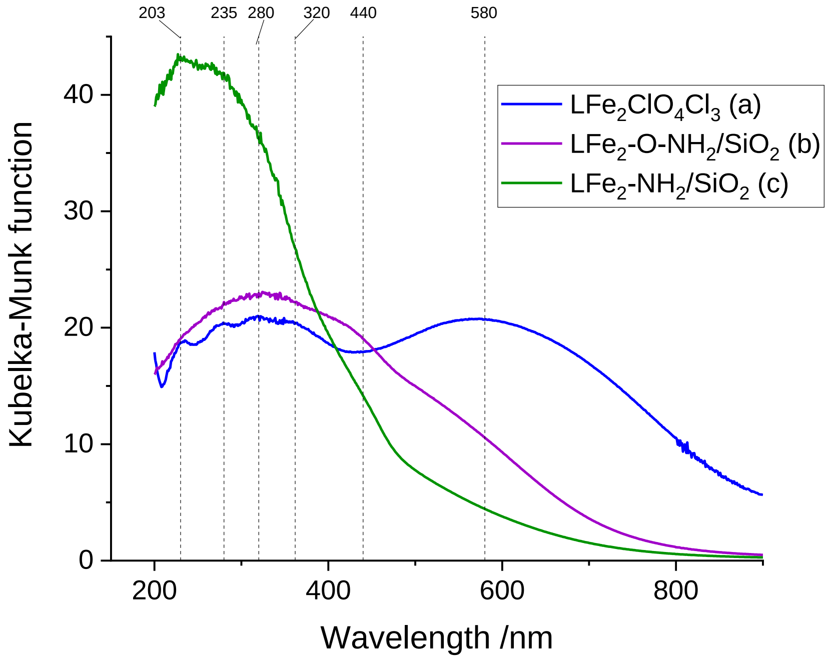

Figure S7. UV-vis spectra of: (a) **$LFe_2Cl_3ClO_4$** (blue line), **(b) LFe_2-O-NH_2/SiO_2** (purple line) and (c) **LFe_2-NH/SiO_2** (green line).

Table S1. Interpretation of proposed models by comparing the experimental and theoretical peak areas from XPS.

S.No		Theoretical peak area ratio	Experimental peak area ratio
1	LFe_2-NH/SiO_2 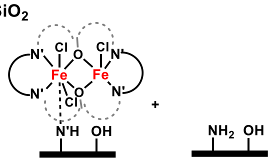 0.25mmol + 0.75mmol	N' = 5X0.25mmol = 1.25mmol<br>N = 0.75mmol<br>N' (H.B) 401.42 eV; N (L.B) 399.52 eV<br>1.25mmol; 0.75mmol<br>ratio= 1.66:1	N' (H.B) 401.42 eV; N (L.B) 399.52 eV<br>1.54; 1<br>ratio= 1.54:1
2	LFe_2-O/SiO_2 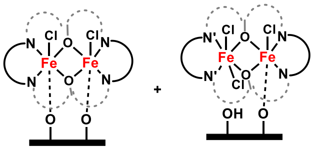	N' = 2<br>N = 6<br>N' (H.B) 401.65 eV; N (L.B) 399.55 eV<br>2; 6<br>ratio= 1:3	N' (H.B) 401.65 eV; N (L.B) 399.55 eV<br>1; 3.5<br>ratio= 1:3.5
3	LFe_2-O-NH_2/SiO_2 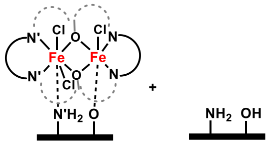 0.27mmol + 0.73 mmol	N' = 3X0.27mmol = 0.81mmol<br>N = 2X0.27mmol = 0.54mmol+0.73mmol= 1.27mmol<br>N' (H.B) 401.38 eV; N (L.B) 399.87 eV<br>0.81mmol; 1.27mmol<br>ratio= 2:3.1	N' (H.B) 401.38 eV; N (L.B) 399.87 eV<br>0.78mmol; 1.26mmol<br>ratio= 2:3
4	LFe_2Cl_4 reference 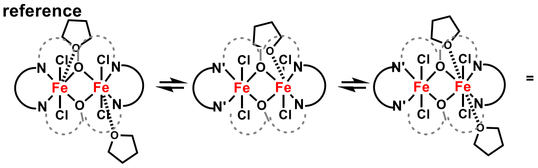	N' (H.B) 401.10 eV; N (L.B) 399.52 eV<br>4; 8<br>ratio= 1:2	N' (H.B) 401.10 eV; N (L.B) 399.52 eV<br>1; 2<br>ratio= 1:2

H.B = higher binding energy; L.B = lower binding energy; **N'**= electron deficient, **N**= electron rich.

Table S2. Atomic composition for solid catalysts based on HR-XPS analysis

S.No	Sample name	ICP analyzing the di-Fe (mmol/g) [%]	Cl (%)	Si (%)	N (%)	Fe (%)	C(%)	O(%)[a]
1	LFe_2-NH/SiO_2	0.25 [0.5]	1.5	23.3	3.0	0.5	21.3	50.5
2	LFe_2-O/SiO_2	0.29 [0.58]	0.7	20.1	1.5	1.0	25.8	50.9
3	LFe_2-O-NH_2/SiO_2	0.27 [0.54]	1.3	15.7	2.7	1.2	33.4	45.6

[a]Oxygen atomic composition reflects contributions of the grafted materials and of the (dominating) signal from the SiO_2 oxygen.

Table S3. LFe_2Cl_4 homogeneous complex molecular determination based on Fe, N, Cl and O atomic composition from HR-XPS analysis[a]

Peak	Area	Factor	N	%AC
**Cl2p**	4348	1.0	4557.6	6.4%
**N1s**	2534	0.5	5078.2	7.1%
**Fe2p$_{3/2}$**	4942	2.0	2516.3	3.5%
**O1s**	3985	0.7	5436.6	7.6%
**C1s**	16884	0.3	53770.7	75.4%

[a]By careful evaluation of atomic composition and XPS N 1s peak area, we are able to determine the molecular structure of the LFe_2Cl_4 homogeneous complex in solid state. N and O atomic concentrations were nearly double to Fe concentration. In addition, the presence of excess eight carbons per complex indicates the presence of two THF molecules per two iron (Note: In LFe_2Cl_4 synthesis THF was used as solvent). On the other hand, the N 1s signal of LFe_2Cl_4 showed two peaks at 399.5 eV and 401.1 eV with a ~2:1 area ratio, indicating the presence of two types of N species in a symmetrical macrocycle. Combining both atomic composition and XPS nitrogen peak area ratio, we speculate that the di-iron complex coexisted in three different forms in the solid state, where each had different portions of residual THF coordinated at the iron centers (see schematic in Table S1A), which leads to non-equivalent nitrogen atoms in a symmetrical macrocycle.

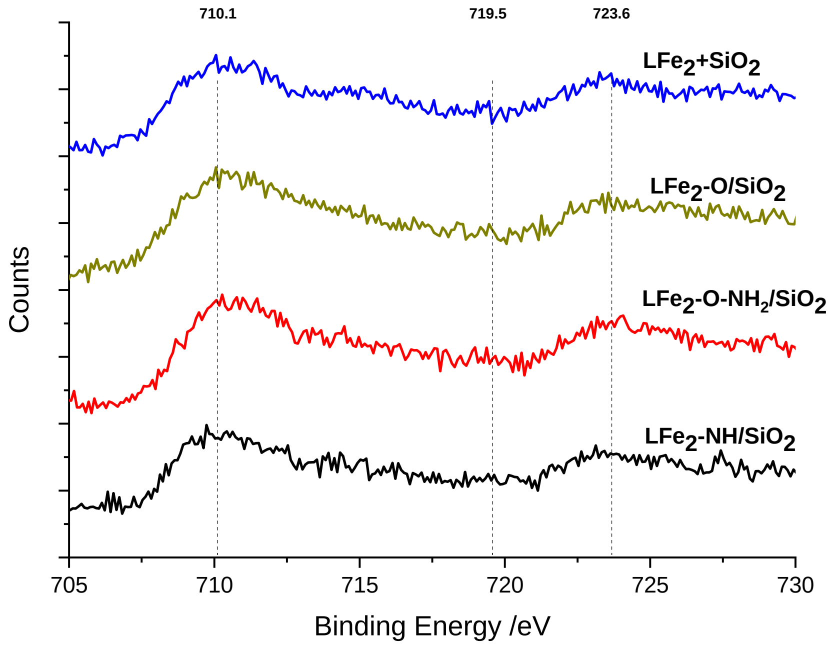

Figure S8. XPS analysis of Fe2p binding energy regions in di-iron (III) solid catalysts. Spectra are offset for clarity.

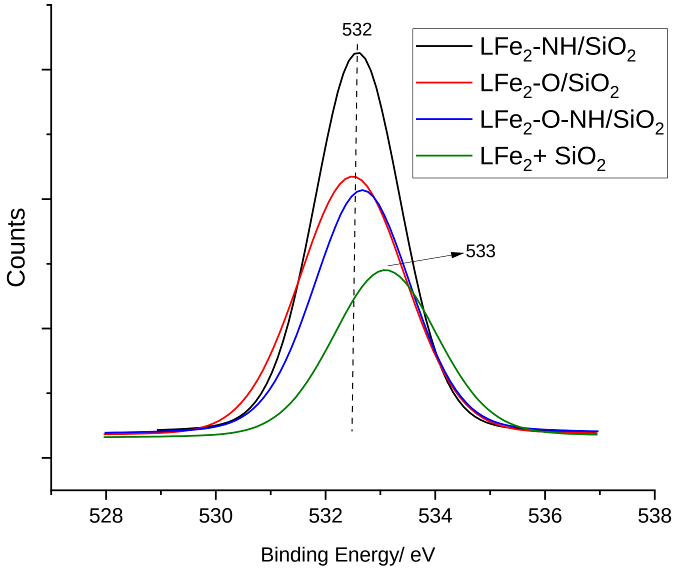

Figure S9. XPS analysis of O1s binding energy regions in di-iron (III) solid catalysts.

Table S4. Conversion of CO_2 to cyclic carbonates using di-iron(III) catalysts at a CO_2 pressure of 15 atm and a temperature of 80 °C.[a]

Entry	Catalysts	Time/h	Yield[b] (g/mg$_{cat}$)	TON[c]	TOF/h^{-1} [d]	% CHC
1	**LFe_2Cl_4**	10	0.41	233	23.3	99
2	**$LFe_2Cl_3ClO_4$**	10	0.46	282	28.2	99
3	**LFe_2-NH/SiO_2**	10	0.36	217	21.7	95
4	**LFe_2-O/SiO_2**	10	0.42	259	25.9	98
5	**LFe_2-O-NH_2/SiO_2**	10	0.62	377	37.7	98
6	**R-LFe_2-O/SiO_2**	10	0.30	182	18.2	96

[a] Reactions carried out under neat epoxide at a loading of [di-iron(III) cat.]/PPNCl/cyclohexene oxide of 1:2:10000. [b] Determined by Measured weight of the isolated cyclic carbonate (yields were normalized per 10 mg of catalyst) . [c] The turn over number (TON) = number of moles of cyclohexene oxide consumed / number of moles of [di-iron(III) cat.]. [d] The turn over frequency (TOF) = TON/reaction period.

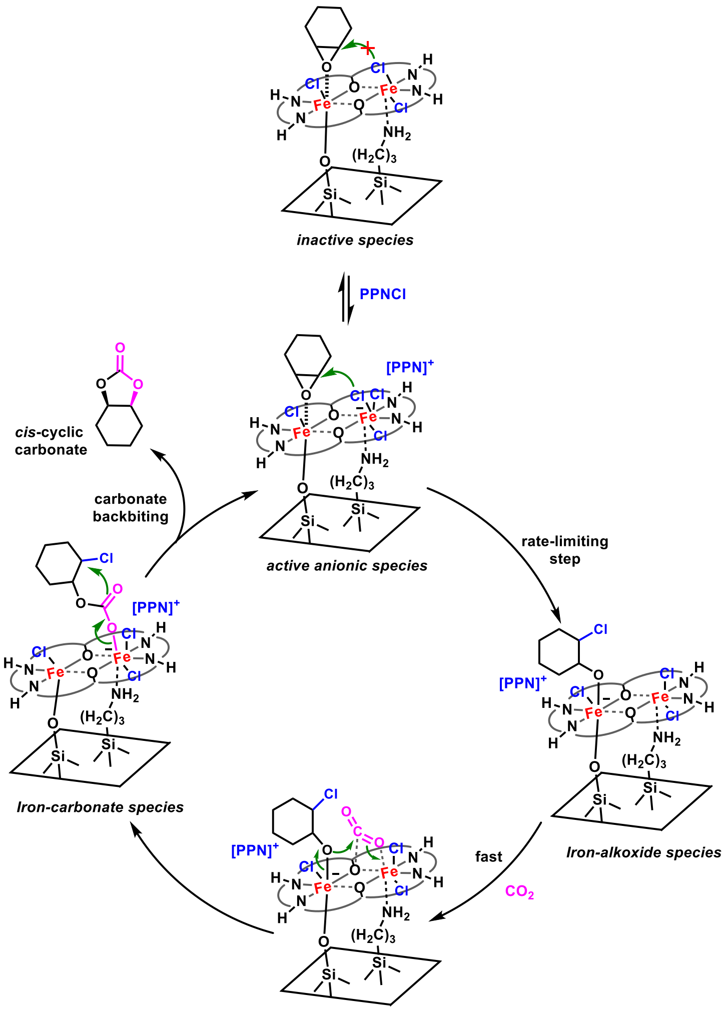

Figure S10. Illustrates the proposed pathways for formation of active anionic species, iron-alkoxide species and iron-carbonate species *via* dual-activation mechanism by two iron metal centers.

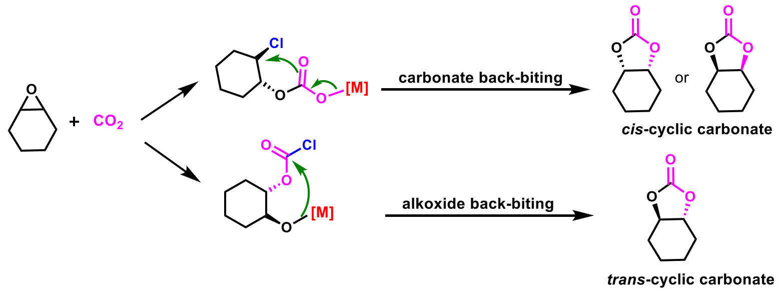

Figure S11. Backbiting reactions from carbonate and alkoxide to form *cis*-cyclohexene carbonate and *trans*-cyclohexene carbonate respectively.[3]

Table S5: Catalytic activity of different heterogeneous catalysts for cycloaddition of CO2 with terminal epoxides

Entry	Sub.	Cat.	Cocatalyst	*T*(°C)	*P*(bar)	*t*(h)	Yield (%)	TON	TOF	Recycling (cycles reported)	Ref.
1	SO	**COF-JLU-7**	TBAB	80	1	12	88	176	15	5	J. Mater. Chem. A, 2018, 6 , 374–382
2	SO	**Eu(BTB)(phen)**	TBAB	70	1	12	98	28	2.3	5	Inorg. Chem. 2016, 55, 9671–9676
3	SO	**Hf-NU-1000**	TBAB	r.t	1	26	100	52	2	multiple[a]	J. Am. Chem. Soc. 2014, 136, 15861 – 15864
4	PO	**Co-CMP-100**	TBAB	25	1	48	81.5	167	3.5	11	Nature Communications, 2013, 4, 1960.
5	PO	**Co-CMP-100**	TBAB	100	30	1	98.1	201	201	22	Nature Communications, 2013, 4, 1960.
6	PO	**Co-MON**	TBAC	60	5	12	70	1400	117	NA[b]	J. Mater. Chem. A, 2013, 1, 5517–5523
7	SO	**gea-MOF-1**	TBAB	120	20	6	85	567	94	4	Nature Chemistry, 2014, 6, 673–680.
8	BuGE	**SBA-15-10-7**	-	50	1	24	80	320	13.3	5	Adv. Synth. Catal. 2019,

											361, 345–354
9	SO	**PS-di-Al(Salen)**	-	26	1	20	79	31.6	1.58	4	Chem. Commun., 2009, 2577–2579
10	SO	**LFe_2-O-NH_2/SiO_2**	PPNCl	80	1	24	65	612	25.5	6	This work
11	PGE	**LFe_2-O-NH_2/SiO_2**	PPNCl	80	1	24	89	888	37.0	6	This work

a= reported as multiple (didn't provide number); b= not available

Table S6: Catalytic activity of different heterogeneous catalysts for cycloaddition of CO2 with cyclohexene oxide

Entry	Cat.	Cocatalyst	*T*(ºC)	*P*(bar)	*t*(h)	Yield (%)	TON	TOF	Recycling (cycles reported)	Ref.
1	$SnCl_4$ (0.66)-IL-Br@SiO_2	-	100	15	48	83	NA	NA	5	Chemical Engineering Journal 422 (2021) 129930
2	PS-Cat 5	-	85	10	18	12	NA	NA	11	Green Chem., 2014, 16 , 1552–1559
3	**YCl@SiO_2**	TBAC	80	10	48	79	24	0.5	5	Catal. Sci. Technol., 2019, 9 , 6152–6165
4	**Amb-OH-I-910**	-	80	10	18	37	17	0.94	5	ACS Sustainable Chem. Eng. 2020, 8, 7993 − 8003
5	**FIP-Im**	-	80	10	48	21	4.2	0.09	6	J. Mater. Chem. A,

										2018, 6 , 9172–9182
6	**Al-iPOP-1**	-	40	10	36	83	830	23	6	ChemSusChem 2017, 10, 2534 – 2541
7	**COP-222**	-	100	1	36	22	NA	NA	15	Chem, 2019 5, 3232–3242
8	**LFe_2-O-NH_2/SiO_2**	PPNCl	80	1	24	0.26 [a]	258	10.8	6	This work
9	**LFe_2-O-NH_2/SiO_2**	PPNCl	80	15	12	0.48 [a]	377	37.7	6	This work

a= Measured yield of cyclic carbonates in grams.

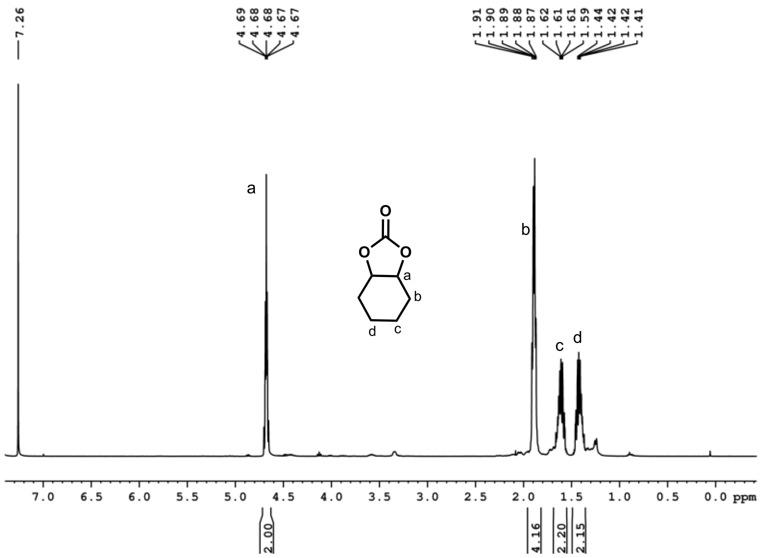

Figure S12. ^{1}H NMR spectrum ($CDCl_3$, 400 MHz, 298 K) of *cis*-cyclic cyclohexene carbonate (CHC) derived by using LFe_2-O-NH_2/SiO_2 and [PPN]Cl at 1atm CO_2. LFe_2-O-NH_2/SiO_2 /PPNCl/epoxide = 1:2:1000, Table 1 entry 5.

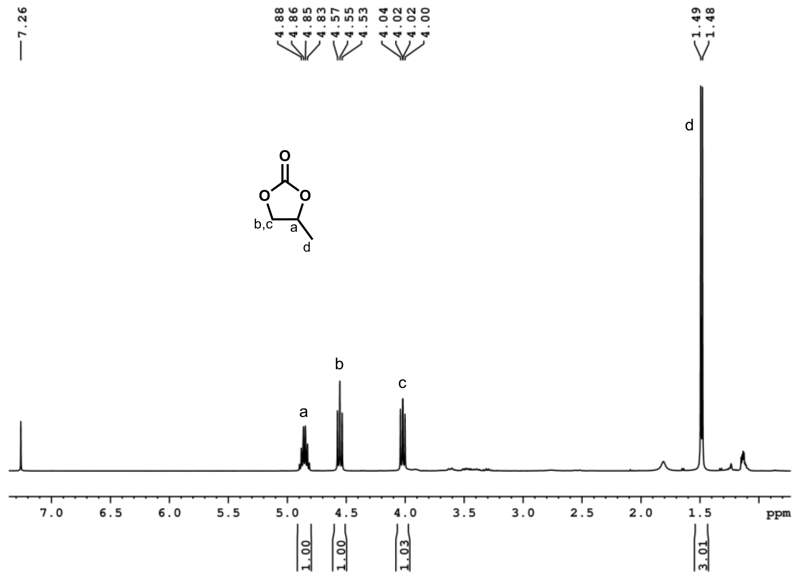

Figure S13. ^{1}H NMR spectrum ($CDCl_3$, 400 MHz, 298 K) of propylene carbonate (PC) derived by using LFe_2-O-NH_2/SiO_2 and [PPN]Cl at 1atm CO_2. LFe_2-O-NH_2/SiO_2/PPNCl/epoxide = 1:2:1000, Table 2 entry 1).

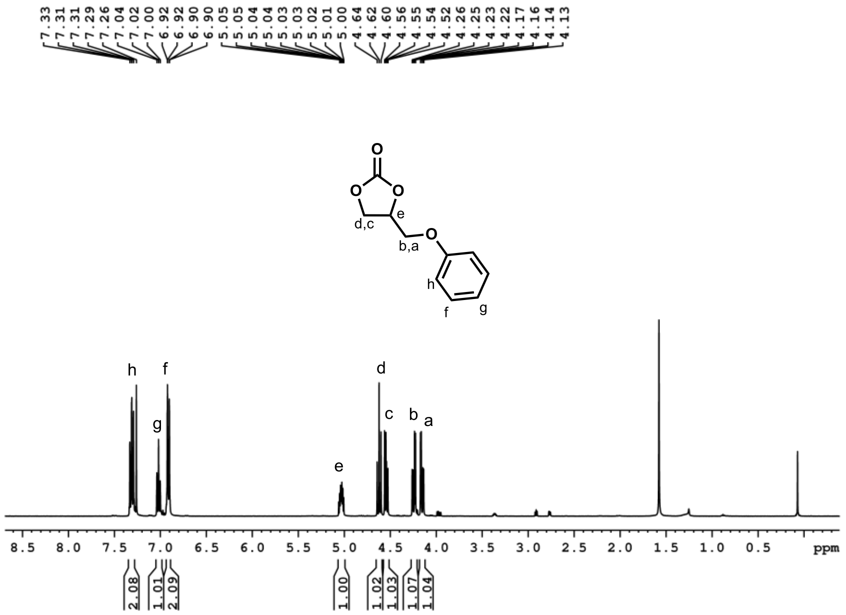

Figure S14. ^{1}H NMR spectrum ($CDCl_3$, 400 MHz, 298 K) of phenyl glycidyl cyclic carbonate (PGCC) derived by using LFe_2-O-NH_2/SiO_2 and [PPN]Cl at 1atm CO_2. LFe_2-O-NH_2/SiO_2 /PPNCl/epoxide = 1:2:1000, Table 2 entry 4.

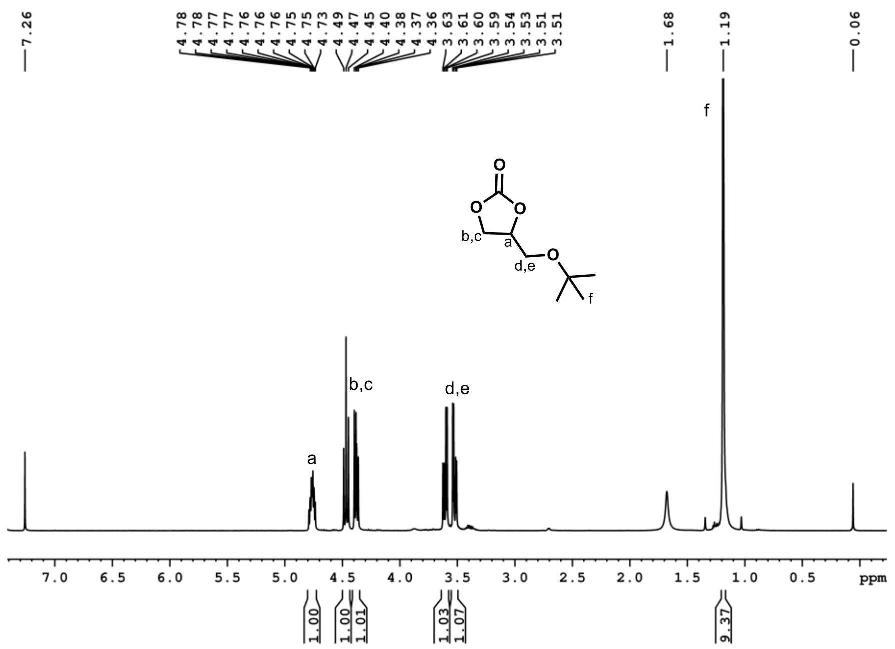

Figure S15. ^{1}H NMR spectrum ($CDCl_3$, 400 MHz, 298 K) of *tert*-butyl glycidyl cyclic carbonate (TGCC) derived by using LFe_2-O-NH_2/SiO_2 and [PPN]Cl at 1atm CO_2. LFe_2-O-NH_2/SiO_2 /PPNCl/epoxide = 1:2:1000, Table 2 entry 3.

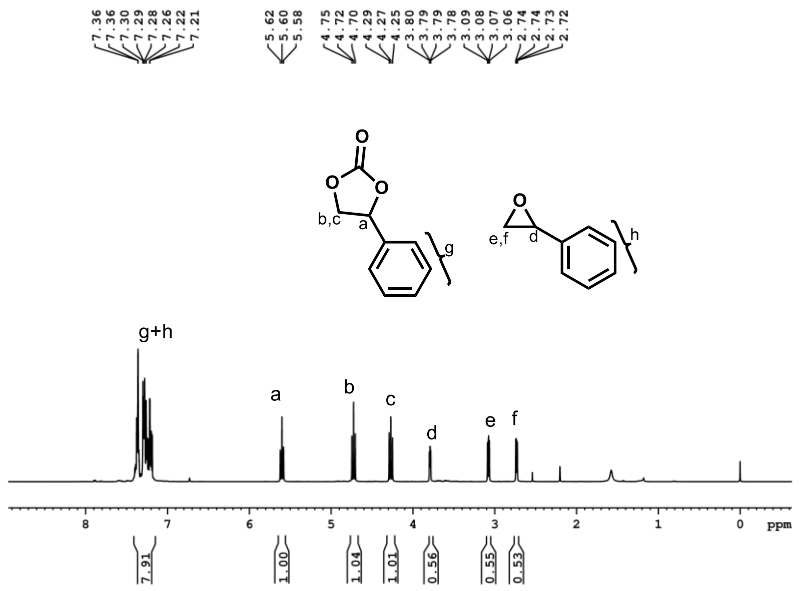

Figure S16. ^{1}H NMR spectrum ($CDCl_3$, 400 MHz, 298 K) of styrene carbonate (SC) derived by using LFe_2-O-NH_2/SiO_2 and [PPN]Cl at 1atm. CO_2. LFe_2-O-NH_2/SiO_2/PPNCl/epoxide = 1:2:1000, Table 2 entry 2.

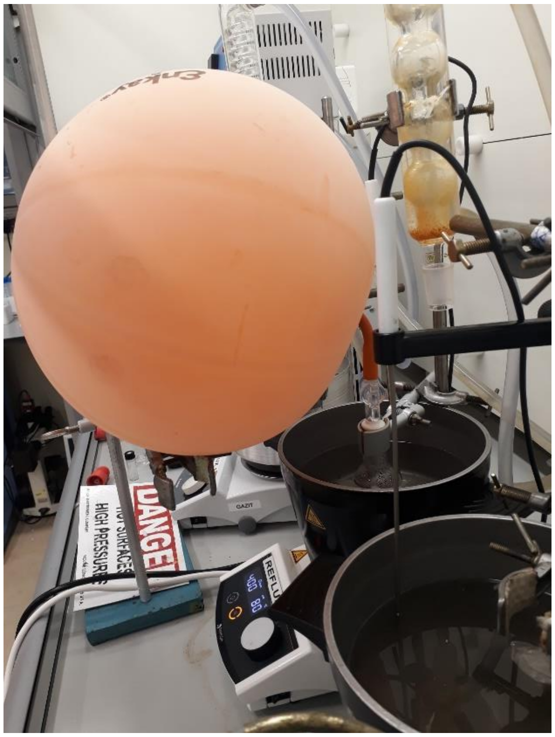

Figure S17. The reaction setup used for the conversion of CO_2 to cyclic carbonate under 1 atm CO_2 pressure.

3. References:

(1) Dutta, B.; Bag, P.; Adhikary, B.; Flörke, U.; Nag, K. Efficient Proton-Templated Synthesis of 18- to 38-Membered Tetraimino(Amino)Diphenol Macrocyclic Ligands: Structural Features and Spectroscopic Properties. *The Journal of Organic Chemistry* **2004**, *69* (16), 5419–5427. https://doi.org/10.1021/jo049787s.

(2) Buchard, A.; Kember, M. R.; Sandeman, K. G.; Williams, C. K. A Bimetallic Iron (III) Catalyst for CO 2/Epoxide Coupling. *Chemical communications* **2011**, *47* (1), 212–214.

(3) Darensbourg, D. J.; Chung, W.-C. Availability of Other Aliphatic Polycarbonates Derived from Geometric Isomers of Butene Oxide and Carbon Dioxide Coupling Reactions. *Macromolecules* **2014**, *47* (15), 4943–4948. https://doi.org/10.1021/ma501004w.
